# Surgery of the Liver

**Published:** 1887-01

**Authors:** 


### SURGERY OF THE LIVER.

In publishing the proceedings of the British Medical Association at Brighton, in May, 1886, the *British Medical Journal*, of the 13th of November, contains a series of papers on hepatic troubles, which deserve most careful consideration by the medical profession. The remarks on hepatic phlebotomy, by Dr. George Harley, open up an entirely new field of investigation, and though he records but one case in which a trocar was used for the purpose of drawing blood from the liver, there are other instances recorded in which punctures of the liver structure have been resorted to with advantage. He states that every reflecting practitioner is aware that neither cupping nor leeching the abdominal parietes can have any direct effect on the amount of blood present in an inflamed liver, from the simple fact that there exists no direct communication whatever between the blood-vessels in the abdominal walls and those of the hepatic organ. It is patently evident, then, that the only way in which either cupping or leeching the hypochondriac region can diminish the amount of blood present in an engorged liver must be simply from the indirect effect the leeching or cupping has upon the hepatic circulation by its reducing the total amount of blood circulating in the patient's body.

He thinks his *confreres* can scarcely feel surprised that, when he had made liver diseases a specialty and began to be called to the bedsides of patients in cases of severe hepatitis, who seemed to be dying from nothing else than the intensity of the engorgement of the liver's blood-vessels, he ventured to suggest to his brother consultants the advisability of trying the curative effects of hepatic phlebotomy. At length he was fortunate enough to meet, in a hopeless case of hepatitis, with ascites and anasarca, a medical brother sufficiently free from the trammels of erroneous clinical teaching, and a patient and husband brave enough to let him try the effects of hepatic phlebotomy as a forlorn hope, when the case had already reached the very gates of death.

Dr. Walker, having rendered the lady insensible with the A. C. E. mixture, he pierced the upper part of the liver from right to left with an eight-inch long trocar of the diameter of between a No. 2 and No. 3 sized English catheter. The normal liver being at least ten inches broad in an averaged-sized woman, and this liver being greatly enlarged—several inches, both laterally and perpendicularly—he felt perfectly safe in running the eight-inch long trocar up to its very hilt. This was done in the hope that during its transverse penetration of the organ it might wound one or more vessels, veins or arteries—it did not matter which—of sufficient calibre to yield a free stream of blood. Twenty ounces of hepatic blood were abstracted without the slightest deleterious result. Exactly eleven weeks after the blood had been abstracted from her liver all the dropsy had disappeared, and the liver might be said to have regained its normal dimensions. Dr. Harley spoke also of puncturing the liver's capsule in cases of chronic congestive hypertrophy with decided benefit in several cases.

In a paper on the surgical treatment of diseases of the liver, by Mr. J. Knowsley Thornton, the reader is interested by the reports of cases in which hydatids of the liver were operated upon successfully, following the notice of a fatal result in a very desperate condition from suppurating hydatids of the liver. He gives the details of a remarkable collection of 412 gall-stones in the liver tissue, with their removal by operation, and suturing the opening in the liver to the opening in the parietes, leaving a small rubber tube in the sac. The wound healed quickly, with hardly any discharge, and the patient was up on the fourteenth day.

A unique operation for opening a liver abscess through a perfectly healthy pleura by sewing the two layers of the pleura together gives a favorable impression of his success in this class of disorders. Mr. Thornton claims that what we have to remember in all these operations is to be scrupulously cleanly in all that we do, and when we cannot destroy a virus, then attenuate it as much as possible and we lessen its power for evil.

Mr. Howard Marsh reports a case in which an abscess of the liver was opened and drained, the edges of the wound being stitched to the abdominal wall. The chief points are that thirty-six ounces of flaky, blood-stained, sero-purulent material escaped from the liver of a boy only 11½ years old, and that he is very well since, but the fistula still discharges clear, bile-stained fluid in small quantities.

Mr. Alfred Willett, after reporting a case of obstruction of the common bile duct, for which he performed the ordinary operation of cholecystotomy, remarks that when consulting with Dr. Gee upon the operation he wished him to undertake, he submitted to him the alternative, in the event which actually occurred, of finding the duct obliterated, of establishing an entero-biliary fistula, but desirous of exposing the woman to as little risk as possible, it was decided to restrict the scope of the operation to external drainage. Nevertheless, reflection has convinced him of the advisability of rendering cholecystotomy, in cases similar to this, a complete operation — complete in the sense of forming an artificial channel between the gall-bladder and the intestine at a point where they lie in close proximity, in order that the biliary secretion shall be discharged after the more normal arrangement into the bowel rather than drain away externally. There are, it appears to him, but two points, from one of which an election must be made: one at or about the junction of the first and second parts of the duodenum, the other where the ascending colon curves and becomes the transverse. It is at one of these two sites that naturally-produced entero-biliary fistulæ are almost invariably found to have occurred. In the group of these cases, nearly thirty in number, collected by the late Dr. Murchison, in about two-thirds the fistulous communication was located in the duodenum and one-third in the colon. There are reasons for and against either of these sites for the operation, so that it would seem difficult to give an absolute preference to either, for a selection of the duodenum would rest upon its appropriateness on physiological grounds, whilst a choice of the colon would be rather surgical, from its close relation here to the gall-bladder. If it fell to his lot to make a choice he would unhesitatingly select the colon, and for these two reasons chiefly: In the first place, he thinks the operation of forming a communication between the gall-bladder and the duodenum would be more difficult in all cases, perhaps impracticable in some, on account of the restricted space at command, the depth at which this part of the duodenum lies and from the nature of the structures—he alludes to the liver particularly—which cover and surround it. In the second place, he submits that the presumed physiological claims for the duodenal site are much less valid than appear at first sight, for microscopic examination of the liver, in cases of long-standing obstruction, show advanced atrophy of the bile-secreting cells; moreover, the fluid found in the dropsical gall-bladder is some evidence of this change; it is of a very low grade, scarcely more than a limpid mucous, and he doubts if any analyst could even call it a "colored imitation" of bile. It may be argued that were the flow of bile into the intestine re-established, functional activity would again take place in the liver cells and a fairly normal bile be secreted, but of this proof is wanting. An objection that may be raised against the duodenal site is that the bile may regurgitate into the stomach and produce troublesome vomiting; but surely bilious vomiting is too common a complaint for its occurrence to be attributable to this altered relation and he fails to see why vomiting should be especially liable to happen. An equally pointed objection may be raised against establishing a direct channel from the gall-bladder to the colon, the change being that now the fæces contain putrefactive germs, and hence the probability is they would find their way into the gall-bladder and along the hepatic duct and excite suppuration in the liver. . . . . He would place the incision in the gall-bladder at its lower part, rather than at the upper, in the expectation that with shrinking of this viscus a tube-like track would be formed with a valvular termination.

The most striking of this series of papers is that of Mr. Lawson Tait on the surgical treatment of diseases of the liver, in which he reports fifty operations performed on the liver, and says all but two have been successful. It is understood that they recovered from the operation, but may have died subsequently from the progress of the disease, whether malignant or otherwise. His table contains seven cases of exploratory operations, thirteen cases of laparotomy and thirty cases of cholecystotomy. It must be admitted, he states, as extremely singular that the cases divide themselves sharply into two groups, one in which the operation is primarily successful, and equally successful in its secondary results; the other in which the operation has a mere temporary success; that is to say, the patient gets over the risks of the operation, but that no satisfactory result in the way of cure is effected, and that these two groups are just as sharply characterized by the complete absence of jaundice in the first and its persistent presence in the second.

The discussion before the Association was limited to Dr. Harley's papers by Drs. Royle, D. Boyer Smith, Culliman, Mr. Millett, Mr. Humphreys, Dr. J. Ewart and Dr. Johnson.

There still remains to be published a paper of Dr. J. McF. Gaston, of this city, on "The Feasibility of Establishing a Communication from the Gall-bladder into the Duodenum," which will be a fit sequel to this important discussion on disorders of the liver.

